# Lipid in Pancreatic Exocrine Cells of Rats Bearing the Walker Tumour

**DOI:** 10.1038/bjc.1972.27

**Published:** 1972-06

**Authors:** E. W. Parry

## Abstract

**Images:**


*Br. J. Cancer* (1972) **26**, 201

### LIPID IN PANCREATIC EXOCRINE CELLS OF RATS BEARING THE WALKER TUMOUR

E. W. PARRY

*From the Department of Histology, The University, P.O. Box 147, Liverpool L69 3BX*

Received for publication February 1972

**Summary.—Exocrine cells of the pancreas of male rats bearing the Walker carcinoma show a striking accumulation of stainable neutral lipid in the form of small aggregated droplets in the base of the cells. In several cases, epithelial cells of small ducts also contained fat. Stainable lipid is sometimes present in cells of the pancreas of normal rats and in rats in which the Walker tumour has failed to grow: lipid in duct cells was confined to tumour-bearing animals.**

It is now generally recognized that tumours bring about changes in chemical composition of many organs of the host animal (Greenstein, 1954). There is also evidence that certain tumours alter the neutral lipid content of some host organs (Boyd *et al.*, 1956; Carruthers and Kim, 1968). The present report describes changes in the quantity of histologically detectable lipid within exocrine cells of the pancreas of rats bearing the Walker tumour.

### MATERIALS AND METHODS

Nine adult male albino rats bearing transplants of the Walker tumour (range of tumour weights 4·5 g to 100 g), formed the experimental group. Control animals comprised one group of 3 animals in which the Walker transplant had failed to grow and a second of 3 normal male albino rats. All animals had access to food and water *ad libitum.* Animals were killed between 10 a.m. and 11.30 a.m. by cervical dislocation under ether anaesthesia. Pancreatic tissue was quickly fixed in cold Baker's formol–calcium for light microscopy. Small blocks were fixed in Palade's (1952) osmium tetroxide fixative for electron microscopy. Formalin-fixed tissues were embedded in gelatine and 10 $\mu$m frozen sections were stained with Sudan 4 in 70% alcohol or with oil Red-O in isopropanol. Sections were then stained with Mayer's haemalum. Osmium-fixed tissues were dehydrated in graded ethanols, cleared in propylene oxide and embedded in Araldite (Glauert, 1965). Sections 0·5–1·0 $\mu$m thick were cut with glass knives and stained with a hot 50 : 50 mixture of methylene blue and azure II in borax for light microscopy. Ultrathin sections, cut with glass knives, were mounted on uncoated copper grids, stained with 0·5% uranyl acetate in absolute methanol, followed by lead citrate (Reynolds, 1963), and examined in a JEM 7 electron microscope.

### RESULTS

Study of 0·5–1·0 $\mu$m Araldite sections first drew attention to the presence of groups of clear or pale green vacuoles situated in the basal regions of pancreatic acinar cells of tumour-bearing rats. Similar structures were seen to stain positively for lipids when frozen sections were stained with the fat stains. Fig. 1 illustrates a typical field from an araldite section of the pancreas of a tumour-bearing rat; the basal vacuoles are numerous and clearly distinguishable from the intensely blue staining apical zymogen granules.

In frozen sections (Fig. 2), the basal vacuoles stain positively for fats. In general, the larger the tumour the more numerous were the fat deposits. In some tumour-bearing animals the epithelial cells of small ducts were also seen to

FIG. 1.—Pancreas of tumour-bearing rat. Numerous groups of clear vacuoles (some indicated by arrows) are present in the basal aspects of many exocrine cells. (Araldite section, metachromatic blue (MB), azure 2 (A2) staining ×480.)

FIG. 2.—Pancreas of tumour-bearing rat showing lipid deposits (black) in exocrine cells. (Frozen section stained with Sudan 4; photographed using green filter ×480.)

FIG. 3.—Pancreas of tumour-bearing rat. Many lipid aggregates are shown in exocrine cells (arrows). In the centre of the field two uniting small tributaries of the duct system also show vacuolation similar to that of lipid on exocrine cells. (Frozen sections confirmed the presence of stainable lipid in some ducts of tumour-bearing animals.) (Araldite section, MB–A2 staining ×1200.)

FIG. 4.—Tumour-bearing rat pancreas illustrating the appearance of small lipid aggregates (L). Some of the components of the aggregates have a dense boundary with the cytoplasm along part of their periphery (arrow). Continuities, in the form of narrow bridges of lipid, are seen between the individual droplets forming the aggregate. Dense zymogen granules (Z). (Electron micrograph, uranyl acetate and lead staining $\times$ 10,000.)

FIG. 5.—Pancreas of rat in which the tumour transplant failed to grow. Contrast the very scarce lipid droplets (arrows) with the abundance of lipid shown in Fig. 1. (Araldite section MB–A2 stain $\times$ 480.)

FIG. 6.—Normal rat pancreas. Arrows indicate a few small lipid aggregates. (Araldite section MB–A2 stain $\times$ 480.)

contain lipid deposits; this phenomenon is illustrated in a "thick" Araldite section (Fig. 3). Electron microscopy shows the basal accumulations to have the characteristic features of lipid, and that although occasional droplets had a dense line around part of the periphery (Fig. 4), they were not separated by detectable membrane from the surrounding cytoplasm. Thus they may be regarded as true lipid droplets.

Control animals in which growth of the Walker tumour had failed to occur showed only occasional deposits of lipid (Fig. 5). Similarly, in normal animals, lipid deposits were very rare (Fig. 6). In neither group was lipid ever detected in duct epithelium.

Despite the fact that the deposits of lipid were large in many cells of tumour-bearing animals, no examples of coalescence to form a single large lipid droplet were seen, although narrow bridges between the individual members of an aggregate were often seen (Fig. 4).

### DISCUSSION

The findings indicate that in normal rats, occasional exocrine cells of the pancreas contain stainable neutral lipids in a characteristic form and position within the cell.

It is also clear that the presence of a medium-sized or large Walker tumour is associated with a great increase in the amount of this lipid. Carruthers and Kim (1968) have shown that the Walker 265 tumour in its carcinomatous form decreases muscle neutral lipids and increases liver neutral lipids. These effects were considered highly specific for this tumour, as other tumours of mammary origin, including the carcino-sarcomatous form of the Walker tumour, failed to bring about these changes. Other authors, however, showed that a Walker carcino-sarcoma could produce an effect on the neutral lipid content of certain organs of host rats (Boyd *et al.*, 1956). In the present work, the tumour used had the histological characteristics of a carcinoma. Several blocks from 4 separate tumours were stained by haematoxylin and eosin and by Gomori's stain for reticular fibres; no sarcomatous areas were seen. It would be interesting to know whether or not this lipid accumulation in the pancreas occurs in rats bearing other types of tumour, and whether or not it is a phenomenon confined to this species.

It is known that the intestine of tumour-bearing rats shows a loss of weight when compared with the intestine of non-tumour-bearing control animals (Bloor and Haven, 1955). There is also an increase in liver weight in tumour-bearing animals (Medigreceanu, 1910), accompanied by biochemical (Greenstein, 1954) and morphological (Ghadially and Parry, 1965) changes in the organ. To the best of my knowledge the pancreas is not recorded as being altered in animals bearing tumours. The present findings show that the exocrine pancreatic cells of rats bearing the Walker tumour are altered in an easily recognizable fashion. Thus, at least in the presence of this particular tumour type, the pancreas may be included with the gut and the liver as an organ showing a well-marked response to a remote neoplasm.

I wish to thank the North West Cancer Research Fund for financial support. The original Walker tumour was kindly supplied by Dr A. W. Craig, Paterson Laboratories, Manchester. I am grateful to Professor N. M. Hancox for reading and criticizing the manuscript.

### REFERENCES

Bloor, W. R. & Haven, F. L. (1955) The Weight and Lipid Content of the Intestines in Rats with Walker Carcinoma 256. *Cancer Res.*, **15**, 173.

Boyd, E. M., Kelly, E. M., Murdoch, Monica E. & Boyd, C. E. (1956) Lipid and Water Levels in Five Organs of Albino Rats Bearing Walker Carcinosarcoma 256. *Cancer Res.*, **16**, 535.

Carruthers, C. & Kim, U. (1968) The Influence of Transplantable Rat Mammary Carcinomas and the Walker Carcinoma 256 on the Lipid Composition of the Muscle and Liver of the Host. *Cancer Res.*, **28**, 1110.

GHADIALLY, F. N. & PARRY, E. W. (1965) Ultrastructure of the Liver of the Tumour-bearing Host. *Cancer, N.Y.*, **18**, 485.

GLAUERT, AUDREY M. (1965) The Fixation and Embedding of Biological Specimens. In *Techniques for Electron Microscopy*, 2nd Ed. Ed. D. H. Kay. Blackwell: Oxford. p. 166.

GREENSTEIN, J. P. (1954) Chemistry of the Tumour-bearing Host. In *The Biochemistry of Cancer*, 2nd Ed. New York: Academic Press. p. 507.

MEDIGRECEANU, F. (1910) On the Relative Sizes of the Organs of Rats and Mice bearing Malignant New Growths. *Proc. R. Soc. B.*, **82**, 286.

PALADE, G. E. (1952) A Study of Fixation for the Electron Microscope. *J. exp. Med.*, **95**, 285.

REYNOLDS, E. S. (1963) The Use of Lead Citrate at High pH as an Electron-opaque Stain in Electron Microscopy. *J. Cell Biol.*, **17**, 208.


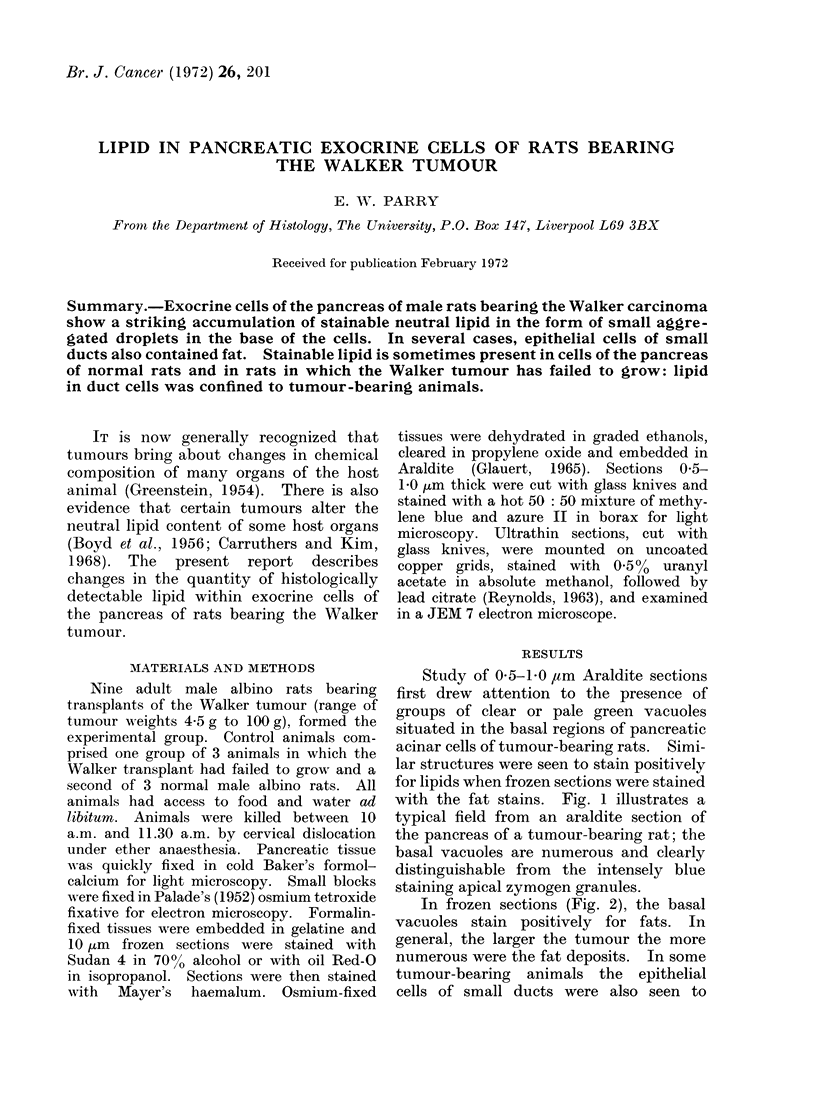

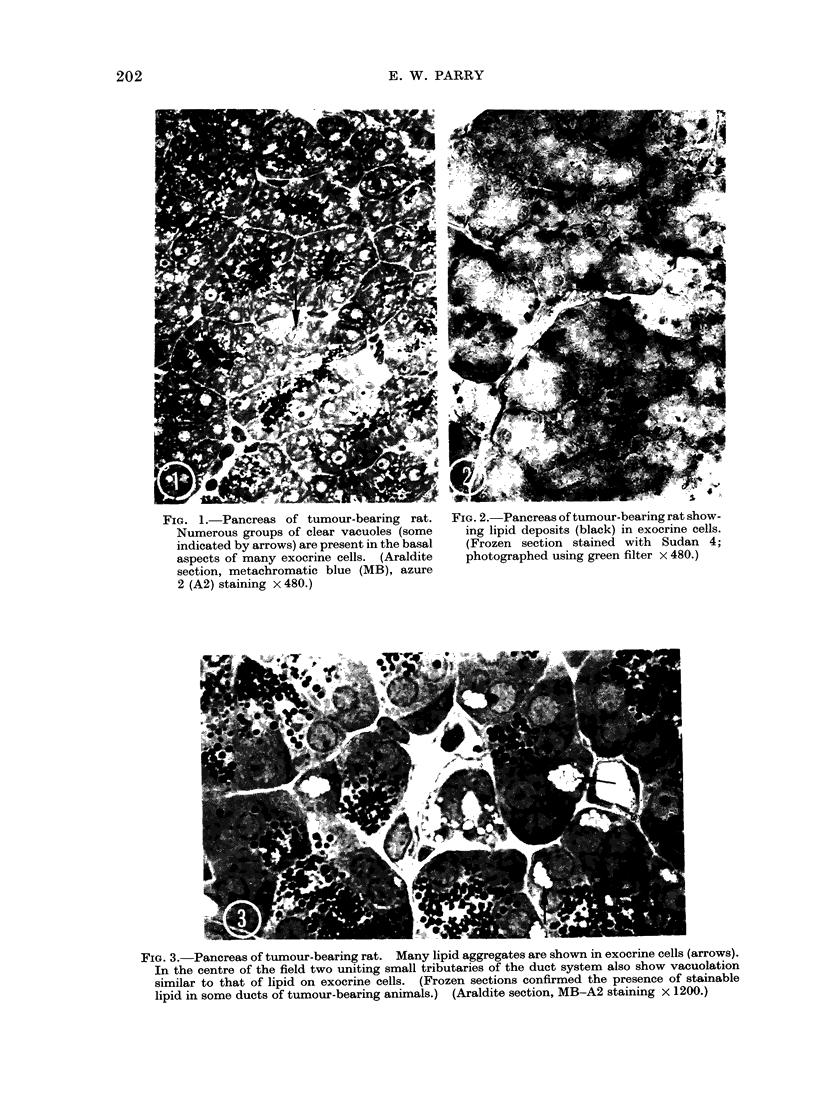

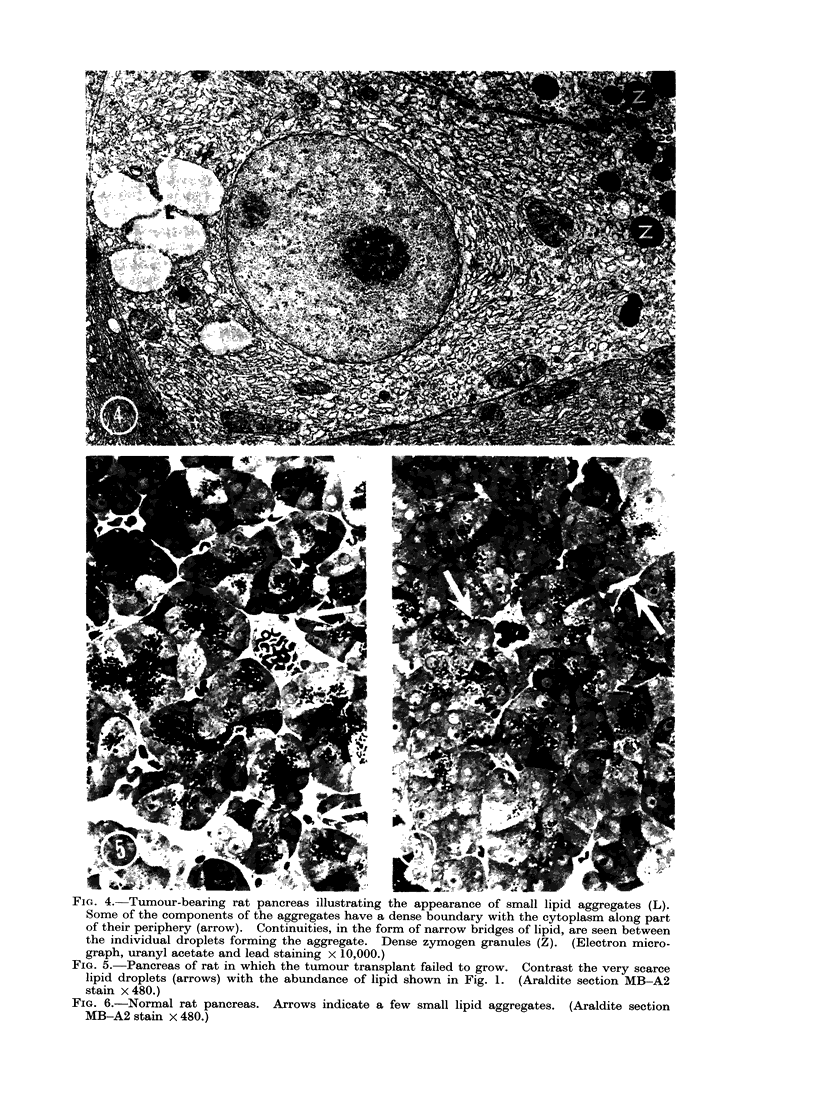

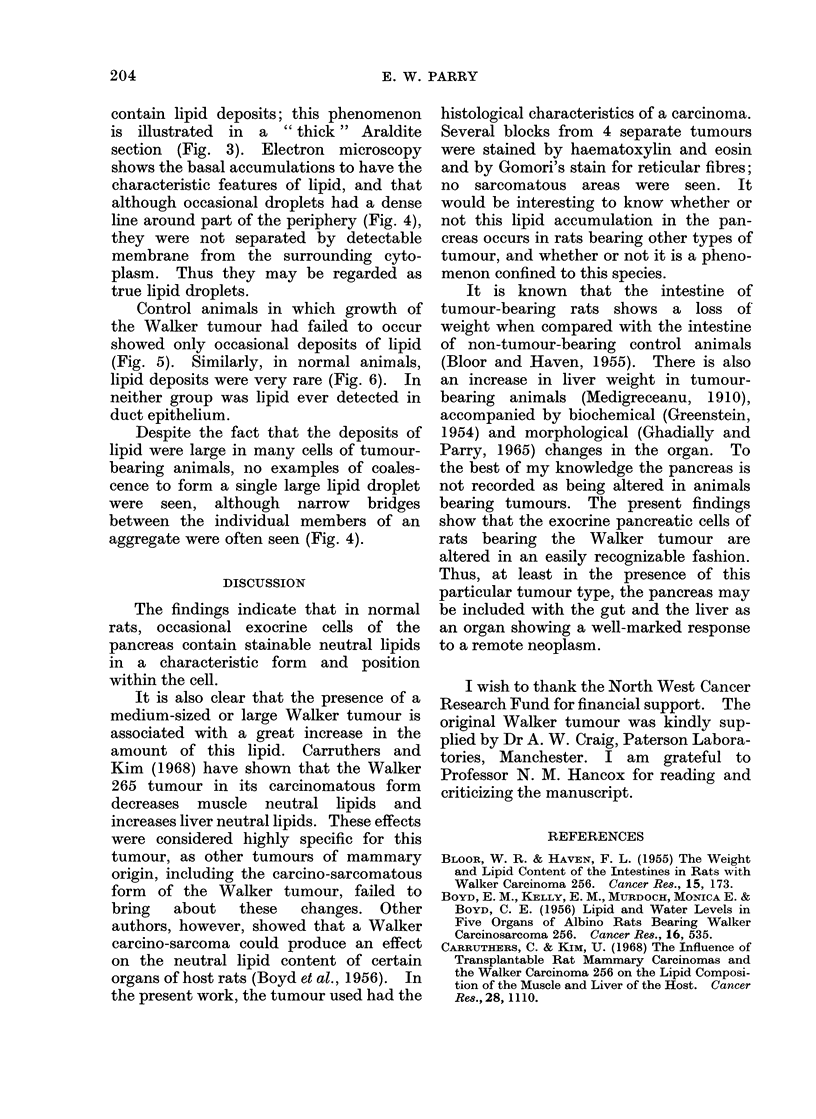

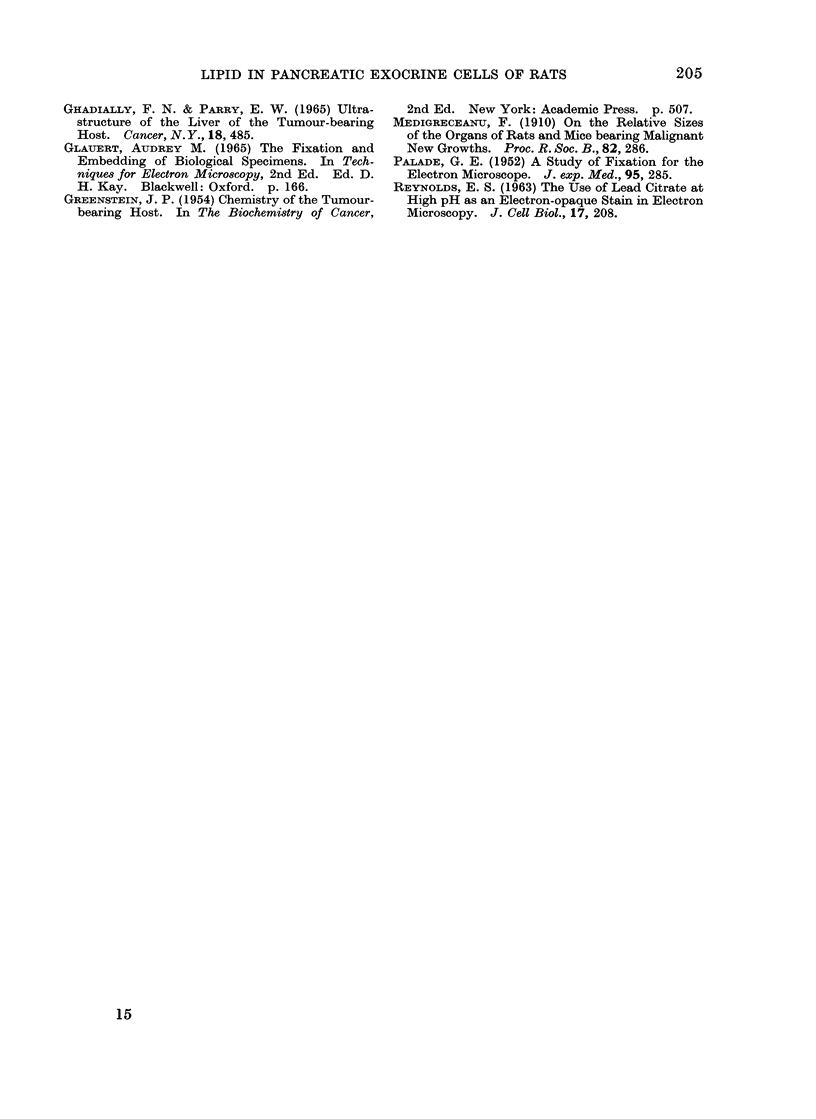


## References

[OCR_00232] BLOOR W. R., HAVEN F. L. (1955). The weight and lipid content of the intestines in rats with Walker carcinoma 256.. Cancer Res.

[OCR_00239] BOYD E. M., KELLY E. M., MURDOCH M. E., BOYD C. E. (1956). Lipid and water levels in five organs of albino rats bearing Walker carcinosarcoma 256.. Cancer Res.

[OCR_00243] Carruthers C., Kim U. (1968). The influence of transplantable rat mammary carcinomas and the Walker carcinoma 256 on the lipid composition of the muscle and liver of the host.. Cancer Res.

[OCR_00252] GHADIALLY F. N., PARRY E. W. (1965). ULTRASTRUCTURE OF THE LIVER OF THE TUMOR-BEARING HOST.. Cancer.

[OCR_00274] PALADE G. E. (1952). A study of fixation for electron microscopy.. J Exp Med.

[OCR_00278] REYNOLDS E. S. (1963). The use of lead citrate at high pH as an electron-opaque stain in electron microscopy.. J Cell Biol.

